# Comparing epidemiologically estimated treatment need with treatment provided in two dental schemes in Ireland

**DOI:** 10.1186/1472-6831-12-31

**Published:** 2012-08-16

**Authors:** Helena Guiney, Patrick Felicia, Helen Whelton, Noel Woods

**Affiliations:** 1 Oral Health Services Research Centre, Cork Dental School and Hospital, University College Cork, Cork, Ireland; 2 Centre for Policy Studies, University College Cork, Cork, Ireland

**Keywords:** Need, Treatment provided, Extractions, Restorations, Dentures, Proportion of treatments, Mean number of teeth, Survey data, Administrative data

## Abstract

**Background:**

Valid estimation of dental treatment needed at population level is important for service planning. In many instances, planning is informed by survey data, which provide epidemiologically estimated need from the dental fieldworkers’ perspective. The aim of this paper is to determine the validity of this type of information for planning. A comparison of normative (epidemiologically estimated) need for selected treatments, as measured on a randomly-selected representative sample, is compared with treatment actually provided in the population from which the sample was drawn.

**Methods:**

This paper compares dental treatment need-estimates, from a national survey, with treatment provided within two choice-of-dentist schemes: Scheme 1, a co-payment scheme for employed adults, and Scheme 2, a ‘free’ service for less-well-off adults. Epidemiologically estimated need for extractions, restorations, advanced restorations and denture treatments was recorded for a nationally representative sample in 2000/02. Treatments provided to employed and less-well-off adults were retrieved from the claims databases for both schemes. We used the chi-square test to compare proportions, and the student’s t-test to compare means between the survey and claims databases.

**Results:**

Among employed adults, the proportion of 35-44-year-olds whose teeth had restorations was greater than estimated as needed in the survey (55.7% vs. 36.7%;p <0.0001). Mean number of teeth extracted was less than estimated as needed among 35-44 and 65+ year-olds.

Among less-well-off adults, the proportion of 16-24-year-olds who had teeth extracted was greater than estimated as needed in the survey (27.4% vs. 7.9%;p <0.0001). Mean number of restorations provided was greater than estimated as needed in the survey for 16-24-year-olds (3.0 vs. 0.9; p <0.0001) and 35-44-year-olds (2.7 vs. 1.4;p <0.01).

**Conclusions:**

Significant differences were found between epidemiologically estimated need and treatment provided for selected treatments, which may be accounted for by measurement differences. The gap between epidemiologically estimated need and treatment provided seems to be greatest for less-well-off adults.

## Background

Valid estimation of the dental treatment needed at population level is important for service planning. In many instances, planning is informed by survey data, which provide epidemiologically estimated need from the dental fieldworkers’ perspective. The aim of this paper is to determine the validity of this type of information for planning. Normative (epidemiologically estimated) need for selected treatments, as measured on a randomly-selected representative sample, is compared with the treatment actually provided in the population from which the sample was drawn.

Commonly defined as the ‘capacity to benefit’ [1], need is considered a key variable in explaining differences in utilisation of health services [2]. Four models of dental needs have been suggested: professionally-defined need (normative or evaluated need), perceived need, expressed demand, and realistic need [3]. The dimension of need recorded by clinicians in epidemiological surveys is ‘normative need’, defined by Bradshaw (1972) as that “which the expert or professional, administrator or social scientist defines as need in any given situation. A 'desirable' standard is laid down and is compared with the standard that actually exists - if an individual or group falls short of the desirable standard then they are identified as being in need” [4]. It is regarded as most accurate when determining the need for simple dental restorations [5]. Evaluated need “represents professional judgement about people’s health status and their need for medical care” [6]. Normative (epidemiologically estimated) need does not capture the patients’ perspective. Translation of need to treatment provided requires action on the part of the patient. This action is the accumulation of a number of behavioural factors initiated by the patients’ recognition or awareness of the problem. Perceived need, reported by individuals, explains care-seeking behaviour, while evaluated need describes the type and amount of treatment that will be provided following assessment of need by the health care provider and consultation with the patient to determine their wants [6].

The two main arrangements that exist for dental care provision for adults in Ireland are the Dental Treatment Benefit Scheme (DTBS) under the Treatment Benefit Scheme operated by the Department of Social Protection, and the Dental Treatment Services Scheme (DTSS) operated by the Health Services Executive. The DTBS, established in the 1952 Social Welfare Act, is a social insurance arrangement through which individuals make Pay-Related Social Insurance (PSRI) contributions; both the employee and employer contribute. Employed adults and retired people, who have sufficient social insurance contributions, and their spouse/partner, were, until 1^st^ January 2010, entitled to receive an annual oral examination, and prophylaxis, every six months, free of charge. In addition, they were entitled to other treatments (such as fillings, extractions and dentures) at a subsidised rate as often as required; prices for some treatments were fixed while others were set by the dentist. The proportion of the cost paid by patients ranged from 31% for X-Rays and surgical extractions (prices set by the Department of Social Protection) to approximately 96% for crowns (prices set by the dentist). This scheme is now restricted to one free annual oral examination.

The DTSS was introduced on a phased basis between 1994 and 2000, beginning with priority for routine and denture treatment for 65+ year-olds, and emergency pain relief for all eligible adults. It provided free dental care to medical card holders until April 2010, when priority was given to emergency dental care with a focus on relief of pain and sepsis. Most individuals aged 16 years or over obtain a medical card if their income is below a certain level, if the cost of meeting medical needs causes a person financial hardship, or if they have an entitlement under EU regulations. The aim of establishing the DTSS was to provide dental services to adult medical card holders to improve their oral health [7]. A greater range of treatments were provided in the DTBS (employed and retired adults) than the DTSS (less well-off adults).

One of the approaches to measuring the impact of these schemes on the oral health of the population is through periodic national surveys of adult oral health. Many surveys add a clinical estimation of treatment need to the record of oral health status, where the examiner first records the condition of the teeth and then records any treatment required (in their clinical opinion). The examining dentist does not have recourse to diagnostic aids such as radiographs to support the clinical examination. This is recognised to be an underestimate, the extent of which is unknown. Analyses of such epidemiological data are used in planning and evaluating services in many countries. Given the methodological difference in the clinical examinations, the frequently low response rate to such surveys, and the fact that evaluated need depends on service utilisation, the validation of these data in estimating treatment need should be explored.

Other studies have compared epidemiologically assessed treatment need with treatment provided within one service [8-10]. However, there are no reports of comparisons between epidemiologically estimated treatment need and treatment provided in two schemes serving different socioeconomic groups in the same population assessed in the same epidemiological survey. In this paper, need for oral health services, as estimated for a representative random sample in a national survey of adult oral health, is compared with actual treatment statistics derived from two dental schemes serving different sectors of the same population. The aim of this paper is to compare epidemiologically estimated oral health treatment need, with treatment provided, as measured from administrative databases, for selected treatments. The comparison is undertaken for two schemes targeted mainly at employed and less well-off adults. The relative relationship between epidemiologically estimated need and treatment provided for employed and less well-off adults is explored.

## Methods

### Estimation of treatment provided

The Department of Social Protection maintains databases of claims for all dental treatments provided under the DTBS, and the Health Services Executive maintains a database of all dental treatments provided under the DTSS. Table 1 presents the key features of each scheme. Following data cleaning and restructuring, use of these databases enabled an analysis of the treatment provided to employed (and retired adults) and less well-off adults respectively. The observations in the DTBS databases were originally arranged as one entry per claim (a dentist could claim for treatments on up to 12 teeth in one form), and the observations in the DTSS database were one entry per treatment. Prior to analysis, the databases were rearranged to create new datasets with one entry per treatment for an analysis of the proportion of users who were provided specific treatments, and one entry per person for mean number of treatments provided. To compare treatments provided in the schemes with epidemiologically estimated need, treatments were categorised as extractions, restorations, advanced restorations, and denture treatments, as described in Table 1. Treatments provided between October 2000 and August 2002 were analysed, corresponding to when the survey data were collected. Ethical approval to analyse these databases was provided by the Clinical Research Ethics Committee of the Cork Teaching Hospitals.

**Table 1:** Key features of the two schemes from which data on actual treatment provided was retrieved

	**Scheme 1**	**Scheme 2**
**Referred to as**	**Scheme for employed adults**	**Scheme for less well-off adults**
**Name of scheme**	Dental Treatment Benefit Scheme (DTBS)	Dental Treatment Services Scheme (DTSS)
**Established**	1952	1994
**Payment method**	Fee-per-item	Fee-per-item and some public health service dentists
**Numbers eligible 2002**	1.3 million	1.2 million
**Eligibility**	Employed and retired adults with sufficient social insurance contributions (and their spouses) in Pay-Related Social Insurance classes A, E, H and P	Mainly adults who are unemployed or earning a very low income
**Categories of treatment provided:**		
**Extractions**	Exodontics and surgical extractions	Exodontics and surgical extractions
**Restorations**	Composite fillings, amalgam fillings, white fillings on back teeth/glass ionomers and pin-retained fillings	Composite fillings (six anterior teeth only) and amalgam fillings
**Advanced restorations**	Endodontics, crowns and bridges	Endodontics (anterior teeth only)
**Denture treatments**	Partial dentures, full-upper dentures, full-lower dentures, full-upper and full-lower dentures, and repair or adjustment	Partial dentures, full-upper dentures, full-lower dentures, full-upper and full-lower dentures, and repair or adjustment

Note: For further information visit http://www.welfare.ie/, http://www.citizensinformation.ie/, and http://www.hse.ie/.

### Estimation of epidemiologically estimated need

Epidemiologically estimated need for treatment was recorded by dentists in the 2000/02 Irish national survey of adult oral health [11]. The study of a stratified random sample of 2,888 adults was conducted by the Oral Health Services Research Centre (OHSRC), University College Cork. The three age groups targeted were 16-24 (n = 1,196), 35-44 (n = 978) and 65+ year-olds (n = 714). The sample was weighted by gender, whether or not individuals had medical cards, and age, to be representative of the population as a whole. Weighting was based on estimates of Irish population totals from the Quarterly National Household Survey in the 3^rd^ quarter of 2001. Ethical approval to conduct the study was provided by the Clinical Research Ethics Committee of the Cork Teaching Hospitals.

The study consisted of a thorough clinical oral examination and a detailed interview pertaining to oral and general health, perception of oral health services and oral health related quality of life. The 32 clinical examiners were public service employees. Training in the clinical indices/criteria for the 32 dentists (30 teams) took place at the University Dental School and Hospital, Cork. The fieldwork was conducted between October 2000 and August 2002 in health service clinics. The standard dental operating light was used for the clinical examination, and a portable dental light was used in the case of subjects examined in their own home, however home-based examinations were the exception.

The clinical examiners assessed each subject for caries, periodontal destruction, tooth wear and denture status, and made a treatment-need decision in light of overall dental health status. They were provided with a set of ‘treatment-need’ codes (for the crown and root), and general guidelines on how to arrive at a decision on the treatment required. However, it was emphasised during training and calibration that many treatment plans were possible for each tooth space, and that the examiners’ own clinical judgement was to be relied upon to select which treatment need score applied in individual cases. Individual variations were therefore to be expected, and it was noted that there may be consistent differences among examiners in allotment of treatment need scores.The treatments estimated as needed by the dental fieldworkers were categorised as

· Extractions (due to coronal caries, periodontal disease and root caries, or for other reasons)

· Restorations (to remove caries lesions, repair trauma or replace unsatisfactory fillings in consideration of both function and appearance, and was regardless of number of surfaces involved)

· Advanced restorations (crown, resin bonded bridge or conventional fixed bridge, pulp treatment required due to coronal caries followed by restoration with filling or crown)

· Dentures (partial denture whose major component was plastic or metal, full dentures and repair or adjustment).

‘Other treatment’ was also recorded in the national survey, and this score was used for any other treatment of teeth not already categorised above, and for re-contouring and repairing restorations. However, this was not directly comparable with any description of treatments provided in the claims databases for the schemes so it was excluded from the analysis.

Further information on recording of epidemiologically estimated need is available from the 2000/02 national survey report [11].

Since it was only possible to measure treatments provided to employed and less well-off adults who used the schemes, the analysis of epidemiologically estimated need only included those who were eligible for the schemes and those who said they visited the dentist regularly (as these were more likely to visit during the period of analysis). Survey respondents were asked which dental scheme they could avail of and whether they had a medical card. The estimated need of those eligible for the DTBS (Scheme 1 - employed adults) refers to those who responded “Own Pay Related Social Insurance dental benefit”. Information on age was only available for eligible adults (not their spouses) in the DTBS claims database, therefore the comparison of epidemiologically estimated need and treatment provided excluded spouses who used the DTBS, and, in terms of survey data (epidemiologically estimated need), excluded adults eligible under their spouse’s Pay Related Social Insurance dental benefit. Some employed adults are not eligible for dental benefit and pay privately for all dental treatment; this group were also excluded from the analysis. The estimated need of those eligible for the DTSS (Scheme 2 - less well-off adults) refers to those who said that they had a medical card. Just over three quarters (77.2%) of those who had a medical card realised that they were entitled to treatment under the scheme.

Adults were also asked how often they attended the dentist over the last few years (“every six months or more often”, “every 6-12 months”, “every 12-24 months”, “every two years or less often”, “occasionally” and “never”). In comparing epidemiologically estimated need with treatment provided, we included only those who responded that they attended at least every two years (i.e. regularly, a combination of the first three options) as these would have had a greater likelihood of using the schemes during the observation period (October 2000 to August 2002 – corresponding to when the survey was conducted).

Proportions and means are presented as measures of epidemiologically estimated need and treatment provided. We used the chi-square test with Yates’ correction to compare the proportion of regularly-attending adults eligible for the schemes who were estimated as needing treatments, with the proportion of adults who used the schemes that were provided with these treatments. The unpaired student’s t-test was used to compare mean number of teeth per person estimated as needing treatment in the survey, with mean number of teeth that were provided treatment under the schemes. Differences were considered significant at p <0.05. Results are presented by age group and type of scheme eligible for/used. The DTBS databases were cleaned using Java applications. The DTBS and DTSS databases were arranged for analysis, and output was generated, using SAS® 9.2. Tests were performed using the Graphpad QuickCalcs Website (http://www.graphpad.com/quickcalcs/contingency1.cfm and http://www.graphpad.com/quickcalcs/ttest1.cfm, accessed March 2012).

## Results

The number of adults examined in the survey, and who claimed that they attended the dentist regularly are presented in Table 2, alongside the number of adults who used the schemes from October 2000 to August 2002, by age group and type of scheme eligible for/used. Among 16-24 year-olds, 52.0% of those who were eligible for Scheme 1 (employed adults) said they visited the dentist regularly, compared to 39.2% of those who were eligible for Scheme 2 (less well-off adults).

**Table 2:** Number of adults included in the analysis by age group, scheme, and data source

	**Scheme 1: Employed adults**	**Scheme 2: Less well-off adults**
**16-24 year-olds**		
**Survey total**	198	263
**Survey regular**^**#**^	103 (52.0%)	103 (39.2%)
**Claims data**^⊗^	100,971	58,702
**35-44 year-olds**		
**Survey total**	307	197
**Survey regular**^**#**^	211 (68.7%)	65 (33.0%)
**Claims data**^⊗^	134,198	48,491
**65+ year-olds**		
**Survey total**	65	456
**Survey regular**^**#**^	28 (43.1%)	94 (20.6%)
**Claims data**^⊗^	3,773	59,948

NA = not available. ^**#**^Survey regular: number of adults who said they were regular visitors to the dentist in the survey. ^⊗^Claims data: number of adults who used the schemes from October 2000 to August 2002. The numbers in parentheses refer to regular users as a proportion of the total sample size.

The percentages of adults estimated as needing specific treatments in the national survey, and the percentages of adults who used the schemes who were provided with these treatments in 2000/02 are presented in Table 3 by age group and scheme.

**Table 3:** Proportion of adults with epidemiologically estimated need, and who had treatment provided in the schemes

	**Extractions**	**Restorations**	**Advanced restorations**	**Denture treatments**
**16-24 year-olds**				
**Scheme 1: Employed**				
**Estimated need (survey regular)**	13.9	62.7	9.0	2.2
**Treatment provided (claims data)**	19.7	59.0	4.3	1.0
**Scheme 2: Less well-off**				
**Estimated need (survey regular)**	7.9	46.2	3.0	2.5
**Treatment provided (claims data)**	27.4	62.4	3.1	2.0
	****			
**35-44 year-olds**				
**Scheme 1: Employed**				
**Estimated need (survey regular)**	17.6	36.7	11.2	13.9
**Treatment provided (claims data)**	13.2	55.7	3.9	3.3
		****	****	****
**Scheme 2: Less well-off**				
**Estimated need (survey regular)**	23.4	53.5	12.7	24.3
**Treatment provided (claims data)**	35.0	61.0	3.2	15.2
	*		***	**
**65+ year-olds**				
**Scheme 1: Employed**				
**Estimated need (survey regular)**	15.8	45.0	10.1	29.4
**Treatment provided (claims data)**	20.3	46.1	2.1	13.6
			*	***
**Scheme 2: Less well-off**				
**Estimated need (survey regular)**	32.9	41.1	3.2	52.6
**Treatment provided (claims data)**	28.5	36.3	1.7	42.5

Survey regular: adults who said they were regular visitors to the dentist in the survey. Claims data: adults who used the schemes from October 2000 to August 2002. * p <0.05; **p <0.01; ***p <0.001; ****p <0.0001, where there is a significant difference between the proportion of adults, who attend the dentist regularly, estimated as needing treatment (epidemiologically estimated need) and the proportion of adults using the schemes who had treatment, based on the chi-square test. Statistical analysis was based on unweighted numbers. Both dentate and edentulous adults were used as the denominator as it was not possible to determine dentate status from the DTSS claims database.

Among employed adults, the proportion of 35-44 year-olds for whom restorations were provided was greater than the proportion estimated as needing them in the survey (55.7% vs. 36.7%; p <0.0001). The proportion of 35-44 year-olds provided with advanced restorations was less than the proportion estimated as needing them in the survey (3.9% vs. 11.2%; p <0.0001), and likewise for 65+ year-olds (2.1% vs. 10.1%; p <0.05). The proportion of 35-44 year-olds and 65+ year-olds provided with denture treatments was less than the proportion estimated as needing them: (3.3% vs. 13.9%; p <0.0001) and (13.6% vs. 29.4%; p <0.001) respectively.

Among less well-off adults, the proportion of 16-24 year-olds who had teeth extracted was greater than the proportion estimated as needing them in the survey (27.4% vs. 7.9%; p <0.0001). The proportion of 35-44 year-olds who were provided with advanced restorations was less than the proportion estimated as needing them (3.2% vs. 12.7%; p <0.001). The proportion of 35-44 year-olds who were provided with denture treatment was less than the proportion estimated as needing them (15.2% vs. 24.3%; p <0.01).

The mean number of teeth per person with epidemiologically estimated treatment need and treatment provided for each treatment are presented in Table 4 by age group and eligibility for/use of the schemes. The mean number of teeth needing treatment is dependent on the mean number of teeth present; hence, the latter is also presented for each age group for employed adults. Number of teeth present was not available from the claims database for less well-off adults.

**Table 4:** Mean number of teeth per person with epidemiologically estimated treatment need and treatment provided

	**Number of teeth present**	**Extractions**	**Restorations**	**Advanced restorations**
**16-24 year-olds**				
**Scheme 1: Employed**				
**Estimated need (survey regular)**	28.3 (2.0)	0.2 (0.4)	1.5 (1.7)	0.1 (0.4)
**Treatment provided (claims data)**	29.0 (2.7)	0.3 (0.7)	2.0 (2.8)	0.1 (0.3)
	**			*
**Scheme 2: Less well-off**				
**Estimated need (survey regular)**	27.9 (2.1)	0.1 (0.4)	0.9 (1.4)	0.0 (0.2)
**Treatment provided (claims data)**	NA	0.5 (1.1)	3.0 (3.9)	0.1 (0.4)
		***	****	
**35-44 year-olds**				
**Scheme 1: Employed**				
**Estimated need (survey regular)**	26.6 (3.4)	0.4 (1.3)	0.8 (1.7)	0.2 (0.8)
**Treatment provided (claims data)**	26.6 (4.6)	0.2 (0.7)	1.4 (2.0)	0.0 (0.2)
		****	****	****
**Scheme 2: Less well-off**				
**Estimated need (survey regular)**	24.7 (5.2)	0.6 (1.6)	1.4 (1.8)	0.2 (0.4)
**Treatment provided (claims data)**	NA	0.9 (2.0)	2.7 (3.5)	0.1 (0.4)
			**	*
**65+ year-olds**				
**Scheme 1: Employed**				
**Estimated need (survey regular)**	18.4 (6.8)	0.7 (1.9)	1.0 (1.5)	0.1 (0.4)
**Treatment provided (claims data)**	17.2 (8.7)	0.3 (1.0)	1.1 (1.8)	0.0 (0.2)
		*		**
**Scheme 2: Less well-off**				
**Estimated need (survey regular)**	16.0 (7.1)	2.4 (4.2)	0.8 (1.3)	0.0 (0.2)
**Treatment provided (claims data)**	NA	0.8 (1.7)	1.4 (2.6)	0.0 (0.3)
		****	*	

Survey regular: adults who said they were regular visitors to the dentist in the survey. Claims data: adults who used the schemes from October 2000 to August 2002. NA = not available. Standard deviations are in parentheses. * p <0.05; **p <0.01; ***p <0.001; ****p <0.0001, where there is a significant difference between mean epidemiologically estimated treatment need and mean treatment provided based on the unpaired student’s t-test to compare means. Both dentate and edentulous adults were used as the denominator as it was not possible to determine dentate status from the DTSS claims database.

Mean number of teeth present was similar among employed adults in the survey and claims data, for example among 35-44 year-olds, mean number of teeth was 26.6 for both databases. Among employed adults, mean number of teeth extracted was less than estimated as needed for 35-44 year-olds (0.2 vs. 0.4; p <0.0001) and 65+ year-olds (0.3 vs. 0.7; p <0.05). Among 35-44 year-olds, mean number of restorations provided was greater than estimated as needed in the survey (1.4 vs. 0.8; p <0.0001). Mean number of advanced restorations provided was less than epidemiologically estimated as needed in all age groups.

Among less well-off adults, mean number of teeth extracted per person was greater than estimated as needed in the survey for 16-24 year-olds (0.5 vs. 0.1; p <0.001), and was less than estimated as needed for those aged 65 and over (0.8 vs. 2.4; p <0.0001). Mean number of restorations provided was greater than estimated as needed in the survey for 16-24 year-olds (3.0 vs. 0.9; p <0.0001), 35-44 year-olds (2.7 vs. 1.4; p <0.01) and 65+ year-olds (1.4 vs. 0.8; p <0.05).

## Discussion

Information on the proportion of adults and mean number of teeth with estimated treatment need for extractions and restorations was obtained from a clinical examination conducted as part of an epidemiological national survey, and information on treatment provided amongst matched age groups was obtained from administrative (claims) data. An advantage of using claims databases to measure treatment provided is that the information represents real-world dentistry. Each dentist has his/her own approach to treatment, and patients have different perceived needs and lifestyle preferences. These data represent the true complexity of what occurs daily in dental surgeries [12]. The similarities in mean number of teeth present between the claims data and the survey data for employed adults instil confidence in the representativeness of the survey sample.

As in Wanman and Wigren, this was essentially an evaluation of treatment need from two points of view [10]. The first was a professional assessment on a random sample of employed and less well-off adults who claimed to attend the dentist regularly, and was based on an examination made by independent dentists (epidemiologically estimated need), where their only consideration was the subject’s oral status. The second was the treatments provided to employed and less well-off adults who used the DTBS and DTSS schemes (evaluated need). For the latter, factors such as aesthetics, cost, and patients’ perceived needs and preferences were also considered in treatment planning.

There was a lack of agreement between mean estimated treatment need and mean treatment provided in all age groups, especially among 16-24 and 65+ year-old less well-off adults and 35-44 year-old employed adults. Although treatment provided was greater than estimated need in some cases, it is important to note that the dentist providing the service had recourse to radiographs, and could therefore offer a more thorough clinical examination than for the national survey. In the national survey, estimated need for advanced restorations included endodontics, crowns, bridges and veneers. However, the only advanced restoration covered by the DTSS (the scheme for less well-off adults) was endodontic treatment for anterior teeth. As endodontics for other teeth were not covered, these less well-off adults may have chosen extraction of compromised teeth rather than incur the expense of advanced restorations. This may explain why the proportions of less well-off 16-24 and 35-44 year-olds who had teeth extracted, was greater than estimated as needed. In addition, Millar and Locker [13] found that people in low-income households were less likely than those in high-income households to mention preventive reasons for visiting a dentist. Extractions have been found to be more likely when the reason for a visit is pain [14], whereas visiting the dentist for a check-up, instead of when in need or pain, is associated with increased retention of natural teeth [15].

There are two possible measurement reasons for the gap between estimated need for restorations and the mean number of restorations provided in the schemes. First, mean need for restorations was calculated regardless of number of surfaces involved, however, patients may have been provided with restorations on different surfaces at several visits to a dentist during the period of analysis. Second, restoration repair may have been recorded as ‘other’ in the survey (as explained in the methods section) and as a ‘restoration’ in the claims databases.

Other studies also found disparities between assessment of dental treatment need and the treatment actually provided [8-10]. Nuttall [8] found a large discrepancy between need for dental treatment recorded in an epidemiological survey and the clinical treatment that was subsequently provided (for the same subjects) in the General Dental Service in Scotland. He suggests that the results “cast doubt upon the usefulness of the epidemiological survey as a tool for predicting restorative treatment” [8]. Naegele and colleagues found that more teeth were treated by fee-for-service dentists, based on a thorough routine dental check-up, than predicted as needed by salaried dentists (within six months) [9]. Wanman and Wigren [10] also question the validity of epidemiological assessment of treatment needs. They compared professionally assessed treatment need in an epidemiological survey with treatment provided in the Public Dental Service in Sweden, and found a significantly higher frequency of restorative treatments provided than the assessed need, especially among 65+ year-olds [10]. Similar results were found in this study, where more restorations were provided than estimated as needed in the survey, across all age groups.

Clarkson and colleagues suggest that the lack of agreement between what dental epidemiologists observe and the treatment that dentists provide may be due to the more complex nature of treatment decisions made by dentists compared to the diagnostic criteria used in conventional epidemiological studies [16]. According to Sheiham and colleagues, a more realistic assessment of treatment needs should include “the functional and social dimensions of dental disease, and an assessment of the social motivational factors which predispose people towards dental ill health and influence the effectiveness of treatment and health education” [5]. In evaluating need in the Irish national survey, no consideration was given to the patient’s financial situation or whether he/she wanted treatment, whereas both patient and oral health factors were considered in the provision of treatment.

According to Schonfeld, gaps between treatment need and treatment provided may indicate requirements for additional manpower, an increase in productivity of existing manpower, or a change in the pattern of dental care [17]. Grembowski and colleagues suggest that under fee-for-service reimbursement, dentists’ efforts to build financially successful practices may encourage over-treatment [18]. Where dental services are provided at zero monetary cost to the patient, as for less well-off adults in Ireland, there may be an incentive for patients to over-consume or dentists to over-provide treatments. According to Woods [19], if there is evidence of either over-consumption and/or over-provision of services, for particular treatments, or to certain groups, then resources should be diverted from areas of excess provision to groups with greatest need.

Although the gap between epidemiologically estimated need and treatment provided seems to be greatest for less well-off adults, we do not know if the differences are related to dentist or patient factors, and therefore we cannot determine whether over- or under-treatment occurred. As in health care generally [20], variations in dental treatment arise from the interaction between supply and demand, which depend on the preferences and perceptions of both patients and dentists; therefore, any differences are probably due to several factors.

This study is concerned with the relative validity of epidemiologically assessed treatment need for adults using two different dental care delivery schemes, one a ‘free’ service for less well-off adults and the other a co-payment scheme for employed adults. The differences between epidemiologically estimated need and treatment provided could also provide an indication of accessibility to dental services for the three age groups in the two schemes. However, differences by socio-economic groups do not automatically reflect inequities [2]. Those in equal need and with equal opportunities to access health care may not make equal use of those opportunities. Nonetheless, an unacceptable reason for differences in use of health care would be that some individuals may be less capable of taking advantage of health care services [21].

In accordance with traditional demand theory, demand for oral health depends on its price per unit, constraining income, the price of all other commodities, and the value people place on oral health as a source of consumption benefit [22]. Income level is associated with utilisation of dental care services [23]. Consumers must allocate their income between buying dental care and other commodities [24]. The price of dental care consists of an out-of-pocket payment and other costs such as travel costs, opportunity cost of the time devoted to dental care, and non-monetary costs (such as time and psychological costs) [25]. Sintonen and Maljanen refer to these as the ‘shadow price of dental care’ [22]. Although treatments are provided at a subsidised rate to employed adults in Ireland, people may still feel the cost is prohibitive, especially for advanced restorations such as crowns. This may explain why the proportion of employed adults receiving advanced restorations was significantly less than the epidemiologically estimated need for the 35-44 and 65+ age groups.

Perceived need has been found to be a stimulus for regular attendance [26]. The large gap between mean number of teeth estimated as needing extractions and teeth extracted among less well-off 65+ year-olds could reflect a difference between need and demand for treatment. People may not feel they need treatment (low perceived need), and those found as needing treatment in the survey may not have visited a dentist under the scheme during the period. According to Holm-Pedersen and colleagues, professionally assessed need for dental treatment, based solely on clinical diagnosis, often leads to an “overestimation of the true need for treatment, especially among frail and functionally dependent elderly people, some of whom do not want treatment, either because there is no perceived need or no expressed demand” [3]. Perceived need may be increased or decreased through, for example, health education programs or changing financial incentives to seek services [6]. We should encourage people to visit their dentist for a check-up rather than waiting until they feel pain. Future surveys should include questions on perceived dental treatment needs, as this would provide further insight into the gap between epidemiologically estimated need and treatment provided.

### Limitations

In this study, we compared adults who said they were regular users in a survey with treatment provided to those who used the schemes. Although the most recent national survey of adult oral health in the Republic of Ireland was conducted 10 years ago, by comparing it with utilisation data of the same time, we feel that our findings are still relevant today. We were unable to compare treatments provided to the same people who were examined in the survey; this would have enabled us to measure whether there was unmet need or variance with provision. However, given the confidentiality issues and difficulties obtaining agreements to link survey and administrative data, we feel that the method used in this paper was a valuable alternative.

Another limitation is that we do not know why the treatments were provided, for example, whether a restoration was provided for aesthetic reasons or due to caries. The DTBS and DTSS databases could be improved by the inclusion of a field, on the claim form, for reasons for provision of treatments; this would provide a more accurate indication of dental health. Administrative databases are a largely untapped resource for analysis of treatments provided, and this study demonstrates their utility. Holtz and colleagues recommend that comparisons across survey and administrative data sources be encouraged, rewarded, and funded so that limitations can be reduced or removed [27].

## Conclusions

In conclusion, significant differences were found between epidemiologically estimated need for dental treatments and treatment provided, as measured from administrative databases for selected treatments for services targeted mainly at employed and less well-off adults. Provision of restorations were generally greater than epidemiologically estimated as needed. These variations may be due to measurement differences between survey and administrative data.

The results of this study have implications for dental public policy. One issue is that the gap between need and utilisation seems to be greatest for less well-off adults. However, it is difficult to isolate its causes, which in turn limits the recommendation of appropriate policy responses. We recommend further research to establish the extent to which differences in utilisation between adults eligible for the two schemes are the outcome of different opportunities, or different preferences. The best way of achieving this would be to combine a future survey with administrative data.

## Competing interests

The authors declare that they have no competing interests.

## Authors’ contributions

HG participated in arranging the data for analysis, performed the statistical analysis, and drafted the manuscript. PF arranged the data for analysis and helped to draft the manuscript. NW was principal investigator on the Health Research Board grant, conceived the idea for this paper, provided advice, and contributed to finalising the manuscript. HW was co-applicant on the grant, provided advice, and contributed to finalising the manuscript. All authors read and approved the final manuscript.

## Pre-publication history

The pre-publication history for this paper can be accessed here:

http://www.biomedcentral.com/1472-6831/12/31/prepub
